# Whole Animal Feeding FLat (WAFFL): a complete and comprehensive validation of a novel, high-throughput fly experimentation system

**DOI:** 10.1093/g3journal/jkad012

**Published:** 2023-01-18

**Authors:** Maria D L A Jaime, Ghadi H Salem, Daniel J Martinez, Sean Karott, Alejandra Flores, Cameron D Palmer, Sharvani Mahadevaraju, Jonathan Krynitsky, Marcial Garmendia-Cedillos, Sarah Anderson, Susan Harbison, Thomas J Pohida, William B Ludington, Brian Oliver

**Affiliations:** Department of Embryology, Carnegie Institution of Washington, Baltimore, MD 21218, USA; Laboratory of Cellular and Developmental Biology, National Institute of Diabetes and Digestive and Kidney Diseases, National Institutes of Health, 50 South Drive, Bethesda, MD 20814, USA; Instrument Development and Engineering Application Solutions, National Institute of Biomedical Imaging and Bioengineering, National Institutes of Health, 12 South Dr, Bethesda, MD 20892, USA; Department of Embryology, Carnegie Institution of Washington, Baltimore, MD 21218, USA; Laboratory of Cellular and Developmental Biology, National Institute of Diabetes and Digestive and Kidney Diseases, National Institutes of Health, 50 South Drive, Bethesda, MD 20814, USA; Laboratory of Cellular and Developmental Biology, National Institute of Diabetes and Digestive and Kidney Diseases, National Institutes of Health, 50 South Drive, Bethesda, MD 20814, USA; Department of Physiology and Biophysics, Case Western Reserve University, 10900 Euclid Ave, Cleveland, OH 44106, USA; Laboratory of Cellular and Developmental Biology, National Institute of Diabetes and Digestive and Kidney Diseases, National Institutes of Health, 50 South Drive, Bethesda, MD 20814, USA; Laboratory of Cellular and Developmental Biology, National Institute of Diabetes and Digestive and Kidney Diseases, National Institutes of Health, 50 South Drive, Bethesda, MD 20814, USA; Laboratory of Biochemistry and Genetics, National Institute of Diabetes and Digestive and Kidney Diseases, National Institutes of Health, 50 South Drive, Bethesda, MD 20814, USA; Instrument Development and Engineering Application Solutions, National Institute of Biomedical Imaging and Bioengineering, National Institutes of Health, 12 South Dr, Bethesda, MD 20892, USA; Instrument Development and Engineering Application Solutions, National Institute of Biomedical Imaging and Bioengineering, National Institutes of Health, 12 South Dr, Bethesda, MD 20892, USA; Instrument Development and Engineering Application Solutions, National Institute of Biomedical Imaging and Bioengineering, National Institutes of Health, 12 South Dr, Bethesda, MD 20892, USA; Laboratory of Systems Genetics, National Heart Lung and Blood Institute, National Institutes of Health, 10 Center Drive, Bethesda, MD 20814, USA; Instrument Development and Engineering Application Solutions, National Institute of Biomedical Imaging and Bioengineering, National Institutes of Health, 12 South Dr, Bethesda, MD 20892, USA; Department of Embryology, Carnegie Institution of Washington, Baltimore, MD 21218, USA; Department of Biology, Johns Hopkins University, Baltimore, MD 21218, USA; Laboratory of Cellular and Developmental Biology, National Institute of Diabetes and Digestive and Kidney Diseases, National Institutes of Health, 50 South Drive, Bethesda, MD 20814, USA; Laboratory of Biochemistry and Genetics, National Institute of Diabetes and Digestive and Kidney Diseases, National Institutes of Health, 50 South Drive, Bethesda, MD 20814, USA

**Keywords:** *Drosophila*, high-throughput, feeding behavior, screening

## Abstract

Non-mammalian model organisms have been essential for our understanding of the mechanisms that control development, disease, and physiology, but they are underutilized in pharmacological and toxicological phenotypic screening assays due to their low throughput in comparison with cell-based screens. To increase the utility of using *Drosophila melanogaster* in screening, we designed the Whole Animal Feeding FLat (WAFFL), a novel, flexible, and complete system for feeding, monitoring, and assaying flies in a high-throughput format. Our 3D printed system is compatible with inexpensive and readily available, commercial 96-well plate consumables and equipment. Experimenters can change the diet at will during the experiment and video record for behavior analysis, enabling precise dosing, measurement of feeding, and analysis of behavior in a 96-well plate format.

## Introduction

Non-mammalian model organisms, such as *Drosophila*, have great potential for phenotypic screens (Willoughby *et al.* 2013; Strange 2016; Sonoshita and Cagan 2017). They are inexpensive to grow, have a short life cycle, and have well-developed genetic tools for probing biological pathways (Griffin *et al.* 2014; Venken and Bellen 2014; Korona *et al.* 2017). These features coupled with the evolutionary conservation of biological processes found in higher organisms like humans have made flies a prime model organism (Adams *et al.* 2000; Wangler *et al.* 2017). Additionally, the ethical and logistical drawbacks of mammalian testing can be overcome through the use of non-mammalian models such as *Drosophila* (Freires *et al.* 2017). Thus, there exists a clear need to increase the throughput of non-mammalian models.

Much of high-throughput screening, even in flies, relies on tissue culture cells (Perkins *et al.* 2015; Tschapalda *et al.* 2016; Esner *et al.* 2018; Heigwer *et al.* 2018). However, the organization of cells into tissues and organs is accompanied by regulatory feedbacks that are not present in tissue culture cells. This complexity is why model organisms have been so critical for advancing our understanding of development (Arias 2008). Whole animal models provide additional insights into pharmacology, toxicology, and disease.

However, whole animal models have low throughput relative to cell screens. Screening *Drosophila* can easily be scaled to test thousands of conditions, but cell-based screens can scale to millions of samples (Inglese *et al.* 2007; Attene-Ramos *et al.* 2013). As a result, *Drosophila* can be used for small scale screening in follow-up assays after ultra-high throughput screens (Tschapalda *et al.* 2016; Rimkus and Wassarman 2018), but flies are not optimal as a primary assay at high-throughput scale.

A high-throughput system for screening effects of compounds on flies requires several advances in the way *Drosophila melanogaster* are typically grown and experimented on in the lab. These advances should be centered on several main aims: reduced assay time, a path to eventual full automation, and miniaturization to reduce compound use and reagent costs typical of large-scale screens.

One of the biggest contributors to extended assay time and labor is that flies are collected, treated, and harvested by transferring between vials or plates. Each of these steps requires careful sorting and manipulation of the flies. Additionally, the food flies consume and excrement they produce are all in the same housing, because it is the flies that are transferred rather than bringing the reagents to the flies. It is possible to develop elegant low throughput ways to separate flies from the media, such as floating larvae to the surface for imaging (Willoughby *et al.* 2013), but this does not address the increased time needed for experimentation. Most methods are not readily amenable to automation scales required for primary pharmacological screening.

Individual compounds in screening libraries are often expensive and present in small quantities (Kenny *et al.* 2015). *Drosophila* are typically grown in vials or plates with 1–10 mL of food (Willoughby *et al.* 2013; Markstein *et al.* 2014). These standard vial volumes are prohibitive for screening small molecule libraries. Ideally, media volumes should be in the μL range. CApillary FEeding (CAFE) assays deliver μLs of liquid food to flies using narrow bore tubes inserted in the lids of narrow vials (Ja *et al.* 2007; Deshpande *et al.* 2014). The Expresso assay (Yapici *et al.* 2016) is a modification of the CAFE assay with multiple single-fly feeding chambers, each connected to a sensor bank that delivers and measures the amount of liquid food consumed by sets of individual flies. These liquid feeding methods can provide excellent measurements of media consumption but again do not scale sufficiently. EX-Q (also Con-Ex (Shell *et al*. 2018)) quantifies fly food consumption as precisely and accurately as CAFE by measuring the dye consumed (Wu *et al.* 2020). The EX-Q/Con-Ex approach is amenable to scaling, and we adapted it to our methods.

We developed the Whole Animal Feeding FLat (WAFFL) to enable high-throughput whole animal screening. The WAFFL incorporates the handling advantages of deep-well plates and the low media requirements of CAFE-like systems. To avoid developing many custom components, we used commercially available consumables and equipment, such as 96-well plates, 96-tip pipettes, 96-well silicone mats, clear acrylic lids, 96-well plate centrifuge rotors, and 96-well homogenizers. We made provisions to reduce manual handling of flies with the intent for eventual robotic screening. The 3D-printed WAFFL system uses a set of interlocking components to allow for measurements of food consumption, behavior, and biochemical assays with essentially no manual intervention after the flies are loaded into the system. The limited need for manual intervention lends to the benefit of being able to perform assays in an isolated system. The WAFFL system is easy to use and compatible with a broad range of assays.

To demonstrate the WAFFL system’s capabilities, we performed a variety of tests using aged adult *Drosophila* flies. Fly food consumption was quantified on various diets and nutritional sources. Fly survival in the WAFFL was determined at 48 h and 7 days. We also measured feeding continuously for 24 h using a Raspberry Pi (RPI) camera system we developed, called the Monitoring Unit for Fruit Fly Imaging in 96-well plates (MUFFIN) (https://doi.org/10.1016/j.ohx.2020.e00160).

## Materials and methods

Design-associated print files and software for described components can be found in the supplement for use by academic researchers under a material transfer agreement (MTA) from the Technology Advancement Office (TAO) of the National Institute of Diabetes and Digestive and Kidney Diseases (NIDDK), an institute of the National Institutes of Health (NIH). Interested parties should contact MTA@niddk.nih.gov.

### Media and buffers

Chemically defined food (CDF) was made as described by Lee and Micchelli (2013) by mixing 3 powder master mixes: essential amino acid mix 2.87 g/L for 100 kCal food and 11.49 g/L for 400 kCal food (TD.10473, Envigo Teklad, IN); nonessential amino acid mix (TD.110036) 2.03 g/L for 100 kCal food and 8.12 g/L for 400 kCal food; and basal mix 3.2 g/L for both 100 and 400 kCal food (TD. 10475). Then a 5X carbohydrate mix was added. A 100X slurry of lipids was added for 100 kCal food NS 400X was added for 400 kCal food before the CDF was adjusted to the final volume. We added the tracking dye Sulforhodamine B (SRB) at a final concentration of 0.5 mg/mL (Sigma-Aldrich, St. Louis, MO) to the CDF or PBS depending on the assay.

Conventional flies were reared on Cornmeal, Sucrose, and Yeast Media (agar 10 g/L, cornmeal 27 g/L, sugar 43 g/L, yeast 15 g/L, 2.8 g/L p-Hydroxybenzoic acid methyl ester in 10.8 mL/L of ethanol, from LabExpress). Germ-free flies were reared on 10:5 fly media (10% glucose, 5% yeast extract, Bacto Agar 1.2%, propionic acid 0.42%) Liquid 10:5 fly media (10% glucose, 5% yeast extract) was fed in the WAFFL. Liguid 10:5 fly media with Ex-Q dye was used for tracking the amount of food eaten per fly (10% glucose, 5% yeast extract, 1% w/v erioglaucine dye). Tissue homogenization buffer (THB) was PBS pH 7.4 plus 0.5% v/v Triton X.

### Fly husbandry

The *Drosophila melanogaster* Oregon R (modENCODE, FBst0025211) and Phs-hidY (FBst0024638; Grether *et al.* 1995) fly stocks were obtained from the Bloomington Drosophila Stock center (Cook *et al.* 2010). The *Drosophila melanogaster* Canton S flies were obtained from the Bloomington Drosophila Stock center (RRID:BDSC_64349). Flies were grown on either Cornmeal, Sucrose, and Yeast Media (LabExpress fly media) or Glucose, Active Yeast, and proprionic acid media (10:5 fly media) at 25°C, 60% RH. 5 females and 4 males were placed per vial for Oregon R and approximately 10 female and male flies per vial for Canton S. Oregon R flies were transferred to fresh vials daily and progeny from previous vials were collected for experiments. Canton S flies were transferred to fresh vials every 3–4 days and progeny were selected for experiments. An Oregon R stock with a Y chromosome carrying a Phs-hid insertion was generated (http://flystocks.bio.indiana.edu/Browse/misc-browse/hs-hid˙method.html), by crossing double balancer females with Y-lethal males crossed with Y-lethal males (FBst0024638, Grether *et al.* 1995). We introduced the Y chromosome into the Oregon R background using double balanced 2nd and 3rd chromosomes. The 4th chromosome was not tracked. For our experiments we used Oregon R (FBst0025211) males and virgin females obtained from the Oregon R Y-lethal stock after 37°C heat shock using a water bath for 2 h during third instar. Canton S flies sex were sorted after mating, for female only experimentation. We verified the absence of males during collection of individual flies. When males were present in the vial, none of those flies were used for experiments. This occurred in about 1 out of every 10 heat shocked vials. Newly enclosed flies were placed in batches of 35 per vial for use in experimental assays. Vials contained either CDF (Lee and Micchelli 2013), LabExpress fly media, or 10:5 fly media . Flies were aged for 3–4 days at 25°C 60% RH. After aging on solid food, flies were loaded in the WAFFL (as described below) to proceed with the defined assays.

### WAFFL components

The WAFFL housing plate and adaptors were designed with Autocad 2014 for Mac (Autodesk, San Rafael, CA). We generated the data file using 3D Lightyear 1.5.2 and sent the file to the 3D printer with Build Station 5.5.1 (3D Systems, Rock Hill, SC). For the WAFFL CO_2_ base, 4 Channel Fly Dispenser, bead remover and WAFFL-IERN we used SolidWorks 2018 (Dassault Systems, Waltham, MA) and Insight 9.1 to create the toolpath data file (.CMB file format) and Control Center 9.1 to send the .CMB file to the 3D printer (Stratasys, Eden Prairie, MN).

The WAFFL housing, adaptor, and transfer plates were made by stereolithography printer in high resolution mode (X and Y=0.08 mm, Z=0.05 mm) on a Viper 2Si SLA (3D Systems, Rock Hill SC) from Accura ClearVue resin (3D Systems, Rock Hill, SC). Pieces were post-processed in a glycol/ether bath for 2 h to soften the supports and remove resin residues then soaked in isopropanol for 30 min, and cleaned with a toothbrush and dental files (25 mm size 030). Pieces were dried with compressed air and, in the case of the WAFFL housing plate, we used compressed air to check the 7 openings at the bottom of each chamber. We used an ultrasonic bath (Quantrex, Kearny, NJ) with isopropanol to remove any leftover support resin. Pieces were air dried and then UV cured in a ProCure 350 oven (3D Systems, Rock Hill, SC) for 40 min. The cross sectionally square end of the chambers was smoothed with a square 2.7 mm file (Harbor Freight Tools, Camarillo, CA).

The WAFFL CO_2_ base was made on a PolyJet printer (Eden260VS, 16-micron layers, X/Y-axis: 600 dpi) with VeroClear plastic (Stratasys, Eden Prairie, MN) with a glossy surface finish. The support material (SUP707) was removed in water using a circulating SRS-DT3 tank (CleanStation, Osseo, MN) and air dried. We drilled a hole for CO_2_ input to the hose barb fitting (19.05 mm), which was attached with a locknut fitted with Teflon tape.

The fly dispensers were printed on a PolyJet printer (as above), with Rigur plastic (Stratasys, Eden Prairie, MN) and a matte surface finish. Support material (FullCure SUP705) was removed using an OBJ-01200 water jet (Stratasys, Eden Prairie, MN) and 1 mm metal rods were used to completely clear the holes, followed by air drying. A rubber tubing (1.95 mm inner diameter) was attached to aspirate the flies.

The bases and spacers for the humidity chambers and other simple pieces were made from cast acrylic cut with a PLS6.150D laser (Universal Laser Systems, Scottsdale, AZ). We annealed the liner pieces using a 40AFE-LT, 800W lab oven (Quincy Lab Corporation, Chicago, IL) at 80°C to prevent cracking.

The bead remover was printed by fused deposition modeling with ABSplus plastic using the Fortus 250mc printer (Stratasys, Eden Prairie, MN). The support material was removed in the SRS-DT3 tank (CleanStation, Osseo, MN) filled with 41.6 L water and 950 g NaOH, rinsed in water and air dried. Each of the prongs of the bead remover had 2 thick nickel plated axially magnetized Neodymium magnets (Apex Magnets, Petersburg, WV) of 3.18 mm diameter X 6.35 mm inserted and sealed with Loctite M-31CL epoxy (Henkel Corp., Westlake, OH).

All 3D printing was performed in house at NIH facilities. We estimate the resin cost at ≈$600 per WAFFL housing, adaptor, and transfer plate setup. Due to the decreasing costs of 3D printing, the complete setup should be ≈$1,000 from a commercial vendor, with the essential WAFFL housing unit costing ≈$500.

### Video monitor

The MUFFIN enclosure is composed of a slotted aluminum frame (80/20 Inc., Columbia City, IN), with 3.18 mm thick black cast acrylic covers (Piedmont Plastics, Charlotte, NC) mounted on the frame. The unit has a monitoring chamber for the loaded WAFFL. The remainder of the unit houses the electronic and optical components.

The monitoring arena was assembled from the following: an acrylic base-plate for securing the WAFFL, a near-infrared (NIR) back-lighting subsystem placed directly beneath the base-plate, an array of camera sensors/lenses mounted above the base-plate, and an acrylic partition to isolate the monitoring arena from the other components.

The base-plate had a cutout matched to the dimensions of the well-plate (2 mm clearance). A white translucent acrylic NIR diffuser (Piedmont Plastics, Elkridge, MD) mounted on the bottom side of the base-plate supported the WAFFL in the cutout. The acrylic mounted 2.54 cm beneath the printed circuit board provided NIR light. The custom designed board consisted of an array of LED emitters (Vishay Semiconductors, Malvern, PA) and associated driver circuitry (Linear Technology, Milpitas, CA). The combination of the LED array and diffuser achieved spatially uniform back-lighting for the WAFFL. Above the WAFFL was an array (Fig. 4e) of 24 RPI NoIR camera boards (Raspberry Pi Foundation, Cambridge, UK) placed in a 6×4 grid such that each lens field-of-view was a 2×2 well region within the 96-well WAFFL format. The camera boards incorporate the sensor/lens assembly, electronically connected via a flexible PCB cable and mechanically mounted at the center of the board via an adhesive pad. The physical dimensions of each camera board exceed the dimensions of the 2×2 well region within a 96-well plate. To achieve the desired positioning of the lenses, the adhesive was removed to free the lens, allowing it to be repositioned relative to the camera board on a custom 3D-printed part (partially hidden in the figure to reveal underlying detail of camera board and lens placement). The lens was designed with slots to interleave and secure the camera boards in press-fit cutouts, such that each lens has a perpendicular top-down view of its assigned 2×2 region. This lens-camera board mounting part is held from above by an acrylic plate suspended above the base-plate by stand-offs (McMaster-Carr Supply Co., Elmhurst, IL). Lastly, to isolate the monitoring arena from the other components in the system, an acrylic sheet was mounted perpendicularly between the base-plate and the camera mount acrylic.

In addition to the monitoring arena, the MUFFIN unit housed the electronics needed for the back-lighting subsystem, as well as the video acquisition and transmission: a 12V power transformer (Qualtek Electronics Corporation, London, ON) to supply the illumination PCB, 24 RPI modules which acquired video from the camera boards, USB power ports (Anker, Seattle, WA) to supply the RPIs, a 24-port Ethernet switch (TRENDnet, Torrance, CA) and a 5-port Ethernet switch (Netgear, San Jose, CA) used to transmit the video data from all 24 cameras to a single destination (e.g. an external control computer) via an ethernet cable. The sole output cable from the ethernet switch was connected to a panel-mount ethernet feed-through connector (Amphenol Corp., Wallingford, CT). Fans (MITXPC, Fremont, CA) are mounted on the back-cover for system thermal management. A power strip with 7 receptacles (Tripp Lite, Chicago, IL) was installed for use by all devices requiring AC power. The main power cord of the power strip was stripped and soldered on to a power entry module (TE Connectivity Ltd., Schaffhausen, Switzerland) mounted on the back-cover of the unit. Detailed cost estimates for the MUFFIN are in Salem *et al.* (2020).

The rear cover of the MUFFIN enclosure had a power entry module for a standard AC power cord and an ethernet cable receptacle. The front of the MUFFIN enclosure had a sliding lift door (Fig. 4r) allowing user access and isolation of the monitoring arena.

### Video software

Custom software ran on both the RPI boards and the external control PC in order to stream the videos over a MUFFIN-limited Ethernet LAN for storage on the PC. The Python-based MUFFIN acquisition software, with a graphic user interface (GUI), recorded video from the 24 RPI cameras enclosed in the system (Supplementary Fig. S4). The software interfaces with the RPI cameras over a local gigabit network. The GUI consisted of a video display window surrounded by status indicators and control buttons. The display window was divided into 4 subwindows, allowing the user to view live video from 4 separate groups of wells at one time. Status indicators and controls are located along the right side and bottom of the GUI. The uppermost group of status indicators on the right side showed experiment information (name, start time, end time, and total run time). Below, a section for computer information (Supplementary Fig. S4c) included hard drive space, host PC IP address, and number of connected cameras. The well selection area window (Supplementary Fig. S4d) contained 4 arrays of check boxes to toggle the groups of wells displayed in the video display windows. This section also contained a button to manually start and stop recording (Supplementary Fig. S4e). Finally, the bottom of the GUI contained a group of buttons that controlled the program settings and displayed the current date and time (Supplementary Fig. S4f). From left to right, this section included buttons to close the application, minimize the application, display time and date, add experiment information, and adjust program settings.

The minimum computer requirements for the MUFFIN video acquisition and GUI in our hands were an Intel Core i5-6xxx or equivalent, Microsoft Windows 7, 8 GB RAM, 500 GB internal hard drive, Gigabit wired ethernet port, and USB 3.0 ports.

### WAFFL fly loading

To load flies, we first placed the WAFFL in the CO_2_ base, making sure that the plate was in the correct orientation with well A1 in the top left corner. We placed flies from vials to CO_2_ pads, we gently dispensed flies in the WAFFL using a 4 channel mouth aspiration device or with a brush using the CO_2_ pad. Once the flies were loaded, we removed the WAFFL from the CO_2_ base. We placed either the silicone mat or clear acrylic lid in the correct orientation to cover the WAFFL. Flies were allowed to recover for at least 1 h from the CO_2_. We loaded every other well with either a female or male fly. The 96-well food plate we used for each WAFFL, had a random orientation in terms of whether the well contained CDF (fed condition) or PBS (starved condition).

### Humidity chambers and MUFFIN

Experiments with the WAFFL housing plate were performed in an incubator at 25°C with 80% RH. Housing plates were kept in a smaller chamber within in the incubator (Sterilite 42.5×30.2×17.8 cm) under high humidity conditions (80–90% RH) to reduce evaporation of the small volume (16 μL) of media (i.e. CDF, PBS). A saturated salt solution of 5M Sodium Chloride (Sigma-Aldrich, S6546-4L) was used to maintain humidity between 80% and 90% RH in the smaller chamber. The WAFFL housing plates were seated on top of an acrylic base above the salt solution so that the surface they rested on was even. The MUFFIN was placed in the same incubator at 25°C with 80% RH, so experiments were performed under the same conditions as regular WAFFL screens were done. Relative humidity and temperature were measured with a hygrometer (Traceable Hygrometer/Thermometer Cat # 35519-044 VWR).

### Feeding behavior

We adapted the EX-Q and Con-Ex feeding quantification assays to the WAFFL system (Shell *et al.* 2018; Wu *et al*. 2020).

Flies were sorted by sex then housed for 4 h in vials containing 1× PBS in 1.2% agar. They were transferred into the WAFFL using the 4 fly aspirator tool. The WAFFL was next placed on a feeder plate containing the appropriate food with erioglaucine dye at 1% w/v. Flies were allowed to feed for 2 h. At 2 h the feeder plate was switched to a feeder plate without dye for 4 to 6 h, which allows all the dye to pass through the fly and be deposited as frass on the WAFFL walls. Flies were then transferred to deep-well plates containing solid 10:5 food. The capillary feeder tips on the WAFFL chambers were thoroughly cleaned by repeatedly dipping in 70% ethanol and then wiping with a paper towel. To collect the dye in the frass, the WAFFL plate without flies was then placed into a molded silicon plate that seals the bottom of each chamber. 400 μL of 1× PBS was then added to each well. An adhesive aluminum foil plate seal was used to seal top of the WAFFL. After shaking the plate to dissolve the dye, 100 μL was removed from each well and put into a microplate reader, where absorbance at 630 nm was read. Plates were scored live/dead flies prior to moving to the initial feeder plate as well as after 6 h feeding time. Dead and damaged flies were excluded from the analysis.

### Food ingestion measurement by fluorescence

To estimate the amount of food that a fly ingested in one feeding event and how long it takes to go through the gut we performed a 1 h feeding assay. Flies were loaded into the WAFFL and were either starved with 1× PBS pH 7.4 or fed with non-dyed CDF for 6 h (starved and fed respectively). They were then transferred to a different 96-well plate containing sulfarhodamide B infused CDF (100 and 400 kCal) fed ad libitum for 1 h. The flies were then collected and individually homogenized for fluorescence quantification, as described below, to measure the amount of food ingested. We used 3 replicate plates per treatment. Each plate had 48 males and 48 females, with half of each starved and the other half fed. Flies were scored for survival, and dead flies were not analyzed further. To measure the amount of food ingested, flies were harvested on 1.1 mL well plates (Axygen P-DW-11-C-S) and homogenized in 200 μL of THB with one 5 mm stainless-steel bead in each well. Plates were sealed with clear adhesive film (MicroAmp, Applied Biosystems by Life Technologies). Then flies were homogenized using a TissueLyser II (Qiagen) set at 30 Hz for 1.5 min. The beads were removed with the 96-well steel bead remover (Supplementary Fig. S2g). The plate with the solution of homogenized tissue with THB was centrifuged at 1,000 rpm for 2 min. Supernatant (100 μL) was transferred to a 96-well flat bottom plate to measure fluorescence on a SpectraMax i3X (Molecular Devices). Amount of food in the fly is estimated based on a calibration curve.

### MUFFIN experiments

We loaded the WAFFL housing plate in the same manner as previously described, with each well alternating between a male and female fly. The housing plate was then covered with a clear cast, acrylic lid. We waited 1 h for the flies to wake up after anesthesia and then placed the WAFFL housing plate into a 96-well plate with wells containing either CDF or PBS, the flies remained in this plate for 6 h. The type of media we put into each well was randomized in order to reduce batch effects. Afterward, we removed the WAFFL from the plate and placed it on a plate with every well filled with CDF. This was then immediately placed into the MUFFIN for recording up to 24 h.

### MUFFIN video acquisition

Custom software was developed to run both on the RPI boards and the external control PC to stream the videos over a MUFFIN-limited ethernet LAN for storage on the PC. The videos were processed using specialized Matlab (Mathworks, Natick, MA) code. Processing the video required 2 offline manual annotation tasks. First, 4 points on each well were marked to define the boundaries of the well. Second, for each well, the center of the food cylinder and its radius were marked in the image. Subsequently, each frame of video was read in and custom algorithms detected fly silhouettes in the image. Namely, the image was binarized with each pixel receiving a binary label indicating whether it is a fly pixel (e.g. foreground) or not (e.g. background). Connected component analysis was run on the binary image. Connected component analysis detected contiguous foreground pixels in the image, identifying them as a single connected object. The area and centroid of each connected component were computed. A size-filtering step was done to retain only the biggest connected component in each well region. The retained connected component was deemed as the fly silhouette. 2 measures were computed based on the fly silhouette and its image centroid. The per-frame food-interaction flag was the first computer measure. When the silhouette was fully enclosed in the region of the image corresponding to the food cylinder, the fly was considered to be in close proximity to the food and the food-interaction index was set to 1; otherwise it was set to 0. The next measure computed was the inter-frame distance traveled. Namely, the Euclidean distance between the silhouette centroid in the current frame and the centroid in the previous frame is computed. The resulting distance, measured in image coordinates (image pixels), was used as an activity measure for the fly. Rather than presenting the raw per-frame food-interaction index and distance traveled measures, a single data point was computed to represent the measures for each set of nonoverlapping 2-min intervals. For the food-interaction index, the data point was taken to be the fraction of frames in the 2-min interval for which the food-interaction flag was set to one. For the distance traveled measure, the data point was taken to be the cumulative inter-frame distance for all the frames in the 2-min interval.

### Statistical analysis

Fly survival statistical analysis were performed using GraphPad Prism version 8.4.2. P-values were determined by one-way ANOVA and Tukey’s multiple comparisons correction. An Excel sheet with the MUFFIN video annotated data was generated, grouping flies by sex and feeding condition. To visualize the trends of each sex on each fed condition, the travel distance data were binned on 2-min intervals. The food-interaction measurements are expressed as the proportion of frames for every 2 min of video in which the fly was in the food region circle. To generate Fig. 5b a smoothing process of the data was conducted as follows: the raw data of food interaction from each fly was aggregated into block of consecutive frames either in the food interaction range or outside of it (food interactions = 1 s and nonfood interactions = 0 s). Based on the work done by Itskov *et al.* (2014), we estimated that a fly starved for 6 h might have on average 7 food sips in a feeding burst. If each sip takes on average 0.13 s, there are 0.91 s per feeding burst. This results in 27.3 frames, since we have 30fps×0.91s. Then, we defined the lower limit of consecutive frames required to be considered as time near the food to be at least 27 frames and 10 frames as time away from food. Therefore, consecutive frames not meeting these criteria were considered noise and were removed to merge the large chunks of food interactions and no-food interactions. Once noise was reduced in the datasets, data were grouped by sex, caloric content, and fed condition. The smoothed trend lines were computed with R ggplot2 geom_smooth (https://ggplot2.tidyverse.org/reference/geom˙smooth.html) with a smoothing factor of 0.25. A 2 tailed Wilcoxon signed rank (Mann–Whitney U) tests on pairs of data distributions (R function Wilcox.test) and Benjamini & Hochberg (BH) p-adjustment method was performed in 10 min windows of the raw data from distance traveled and food interactions (15–25, 25–35, 35–45, and 45–55 min). MUFFIN video processing software was written in MATLAB 2017b (MathWorks). Statistical analysis was performed in R version v3.5.0 (http://www.R-project.org). Statistical analysis of relative food content per fly after 1 h feeding was performed in R version v3.5.0 (http://www.R-project.org).

## Results

### System overview

**Fig. 1.**
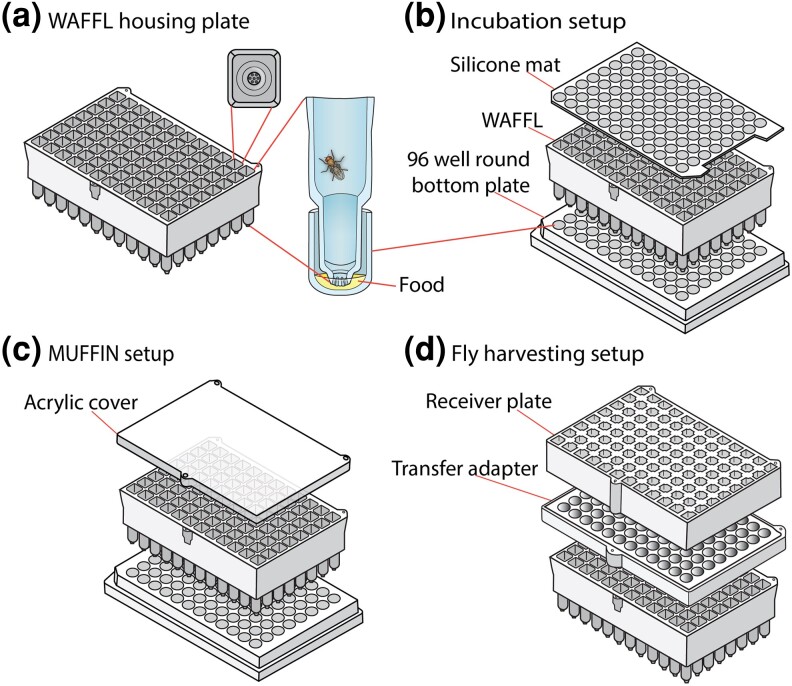
WAFFL apparatus. a) Each WAFFL houses flies in a 96-well plate format and provides food through a capillary in the bottom of the well. b) Food is provided in a 96-well plate that sits underneath the WAFFL, allowing up to 96 parallel experiments in a single WAFFL. The top is sealed with a reusable silicone mat to maintain humidity. c) To visualize fly behavior during experiments, an acrylic cover can be substituted for the silicone mat. d) To transfer flies out of the WAFFL while maintaining their well positions on the 96-well plate, a transfer adapter and receiver plate is used.

We developed the 96-well WAFFL system to rapidly perform high-throughput experiments and deliver essentially any compound or diet in 16 μL of liquid media, enough for 18 h of exposure. We designed a housing plate, in which flies feed through a series of pores at the bottom of each chamber when the housing plate is placed into a standard 96-well microtiter round or V bottom plate with liquid food (Fig. 1a, Supplementary Fig. S1). Because we designed the housing and liquid food plates as separate components, the media can be changed as desired without manipulating the flies (Fig. 1b). One simply prepares a new 96-well plate of food, starvation media, or other test substance, and the housing unit is moved from the old to the new feeding plate. This was designed to allow for a variety of experimental designs, such as the effects of order of delivery to the flies, or the effects of an acute bolus versus chronic exposure.

The WAFFL housing chambers have square cross sections at the top (to maximize living quarters for the flies) and circular cross sections at the bottom (for fitting into standard plates). The top part of the chambers is rectangular cylinders, with the long axis in the Z-plane (8mm×8mm×24.5mm) (Fig. 1a, Supplementary Fig. S1). At the base of the rectangular portion, there is an inner edge that protrudes 0.4 mm from the inner walls (Fig. 1a). Each chamber has a conical protrusion (11.3 mm in length) with a slightly flattened bottom and 7 evenly spaced 350 μm diameter holes (Fig. 1a, Supplementary Fig. S1). The volume of each chamber is 1.6 mL. The housing plate also includes 9 asymmetrically located orifices where steel alloy dowels are fitted and then sealed with clear silicone to allow the housing plate to magnetically attach to the complementary pieces of the transfer system only when the pieces are matched in the correct orientation. This design reduces the chances of mislabeling samples due to plate rotations during the course of an experiment.

The top of the housing plate is compatible with 96-well plate silicone mats and custom acrylic lids (held by dowels) for video monitoring, using the MUFFIN (Figs. 1, 4). This system allows us to see if the flies are interacting with or avoiding the media and allows for scoring behavior or death on treatment.

Flies can be harvested at the end of an assay by the transfer system, which recovers flies for essentially any assay, such as microscopy or homogenization in a 96-well format with stainless-steel beads (Fig. 1d, Supplementary Fig. S1). Many of our assays involve homogenization, and beads can interfere with pipetting the supernatant, so we developed a 96-magnet bead remover (Supplementary Fig. S2g).

A short video showing the use of the WAFFL is available (YouTube https://youtu.be/6yJ4Mq8xIXc and supplementary file).

### WAFFL housing unit

The housing plate is the main element of the WAFFL system (Fig. 1a, Supplementary Fig. S1a). Up to 3 flies can inhabit each of the 96 chambers in the WAFFL, allowing for some pooling to dampen individual variance, but the real strength of the system is the ability to measure those individual differences by using a single fly per chamber. Previous work has shown that 1.1–1.3 mL 96-well plate formats with solid food are effective for screening flies (Willoughby *et al.* 2013; Markstein *et al.* 2014), so the WAFFL housing chamber (1.6 mL) is spacious. The upper portions of the WAFFL plates are square, to provide maximum volume per well while also being compatible with commercially available silicone mats that can be used to seal flies within the WAFFL plates, with or without punctures for gas exchange (Fig. 1b, Supplementary Figs. S1, S2).

The WAFFL is designed to use liquid food. The food access points at the bottom of the WAFFL are critical and were one of the most challenging parts to design (Fig. 1a). We experimented with several different designs focused on the spacing between the WAFFL housing unit, the inside of commercial 96-well plates, and the access ports that allowed the flies to feed. To make a tight fit, we implemented a design with square chambers on the upper portion of the WAFFL that transitioned into a cupular projection at the bottom of the housing unit. This projection fits inside the 96-well plate with 1–2 mm clearance between the wall of the WAFFL housing plate and the well of the food plate. Tighter clearance distances did not provide enough room for liquid in the food plate and there was excessive capillary action (this also depends on the viscosity of the media) between the WAFFL and the food plate, including the spaces between wells. Capillary action exterior to the housing chamber did not allow adequate food access for the flies and resulted in high mortality. More loosely fitting plates constrained flies at the feeding position and increased the volume of media used in each well.

We tested different types of openings for the cupular feeding ports where the media is accessible to the fly. This included proboscis-sized sipping ports and larger openings, giving flies easy access to the media. We observed that when we provided a large opening or many openings at the bottom of the WAFFL plate, flies often became trapped in the liquid media. This did not occur when we reduced the size of the openings and provided a horizontal surface near the feeding port (at the transition from the square to conical shape) where flies can sit. This perch also helped keep flies from falling directly into the feeding area.

Small ports cannot be 3D printed by low-resolution additive manufacturing devices. For our final design, we determined that a high layer resolution of 0.05 mm (Z) and in plane resolution of 0.08 mm (X–Y) was sufficient. In the production model, we created 7 350 μm diameter openings at the slightly flattened terminus of the cupular projections. This arrangement and size provided a good balance of space for the flies to feed, without falling due to the presence of excess liquid.

When considering the material used for printing the WAFFL, we needed to ensure that the housing plate was waterproof so that the liquid in the food plate that we place the WAFFL into was not absorbed by the plate material and could be easily cleaned. The material also needed to be relatively non-reactive with the compounds we might use in assays, biosafe, structurally stable, and not bleed during the printing process as to not alter the specified dimensions. After evaluating and testing the ability of the materials to meet our requirements, we chose Accura ClearVue, a USP (United States Pharmacopeia and National Formulary (USP – NF) plastic designation) class VI capable, transparent, and bio-compatible stereolithography (SLA) resin.

We tested loading flies into the WAFFL as 2nd or 3rd instar larvae or pupae. 1st instar larvae can pass through the feeding ports. We focus herein on adult flies. To facilitate loading of flies into the WAFFL, we printed a custom 4-channel fly dispenser (Supplementary Fig. S2d), which aspirates (by mouth or by vacuum) 4 flies at a time for transfer into the housing plate. To keep the flies anesthetized during loading, we designed and printed a WAFFL CO_2_ base. The housing plate is securely seated in the CO_2_ base and humidified CO_2_ enters through the 7 openings at the bottom of the cupular section of each well. These additional components facilitate faster and more consistent fly loading.

The assembled WAFFL housing plate and food plate can be wrapped with parafilm to minimize evaporation during an experiment longer than 8 h. Additionally, we use plastic boxes to hold groups of plates in a humidified environment. The plates sit in a base above a saturated salt solution selected to maintain a desired (high) humidity level in the chamber (Supplementary Fig. S1h). Groups of plastic boxes are then placed in a larger incubator.

### Harvesting tools and complementary pieces

We wanted to design a method to transfer the flies from the housing plate to a deep-well plate for tissue homogenization and further assays. This can be simply achieved by inverting a fresh plate, inverting the paired plates, and tapping the flies into the new plate. However, flies often became squished by this method. To improve survival and to facilitate eventual robotic implementations, we wanted to transfer to a range of plate types. We also wanted to promote better tracking by maintaining a single plate orientation, which is inverted when flies are simply flipped to a new plate (e.g. well A1 can become A12 or H1). Therefore, we developed a series of intermediate adaptors that retain the orientation of the plate throughout the experiment (Supplementary Figs. S1, S2). As a tracking feature, we added an asymmetric orientation locking system via 9 pairs of steel dowels and magnets in each piece of the system, ensuring a unique alignment of the 3 components of the WAFFL system (housing, receiver plates, and transfer adapter). This eliminated the possibility of rotating plate orientation during processing and subsequently mislabeling the samples. These transfer devices fit in a centrifuge so that anesthetized or frozen flies can be flipped and centrifuged to transfer.

For many experiments, such as RNA-seq library construction, reporter expression, or metabolite measurement, homogenization is required. In order to macerate the flies, we simply added a stainless-steel bead into each well of the deep 96-well plates containing the flies with the selected buffer for use and loaded the assembly into a bead beater. Following homogenization, removal of supernatant with a pipette from the deep-well plate with the 5 mm stainless-steel bead inside each well was difficult and inaccurate, as beads block the pipette tips. To address this, we designed and printed a 96-pronged magnetic device to remove the stainless-steel beads (Supplementary Fig. S2g). The key design challenge was that each of the 96 prongs needed to have a magnet strong enough to grab the bead, but not so strong that the magnets would interact with each other and deform the prong geometry. We chose to encapsulate 2 nickel plated axially magnetized Neodymium magnets (3.18 mm diameter × 6.35 mm thick) in each prong. As configured, the bead remover was able to remove each bead in one swift motion, making the processing of the homogenized fly tissue more efficient.

Frass can be used to measure feeding behavior (Wu *et al.* 2020) or metabolism. We designed the WAFFL Insect Excrement Removal Nano-brushes (WAFFL-IERN) to aid us in removing the frass of the flies that underwent screening in the WAFFL (Supplementary Fig. S2i,j). The WAFFL-IERN allows us to actively measure the amount of food that passed through each fly by adding nonmetabolizable, nonabsorbable tracer dyes to the media and measuring excrement. Measuring excrement has the added advantage of making it possible to assay for a range of poorly studied parameters, such as excreted metabolites, drug clearance, and antibiotic effects on the microbiome.

The main part of the WAFFL-IERN is a handle with 96 micro brushes that align with the WAFFL housing chamber plate (Supplementary Fig. S1i,j). After the food plate and flies are removed, the WAFFL housing plate with excrement is placed into a 96-well plate with a suitable buffer (we have used PBS pH 7.4 + 0.5% v/v Triton) and the WAFFL-IERN is used to manually brush the interior of the WAFFL chambers with a series of up and down movements. The WAFFL-IERN fits into a housing that supports the handle, such that the brushes are unable to reach the bottom of the WAFFL. The WAFFL-IERN and housing is centrifuged to remove liquefied excrement for collection in a 96-well plate. We designed a spacer for use during centrifugation to raise the WAFFL 5 mm from the base of the flat bottom 96-well plate so that the excrement solution does not touch the tip of the cupular wells of the WAFFL.

We also developed a complementary frass harvest method using a molded silicone plate to seal the bottom capillaries of the WAFFL and an adhesive foil to seal the tops of the wells (see Materials and methods). This method is faster and easier than the WAFFL-IERN but in some cases with dried on frass, the WAFFL-IERN may be preferable.

### Fly survival in the WAFFL

**Fig. 2.**
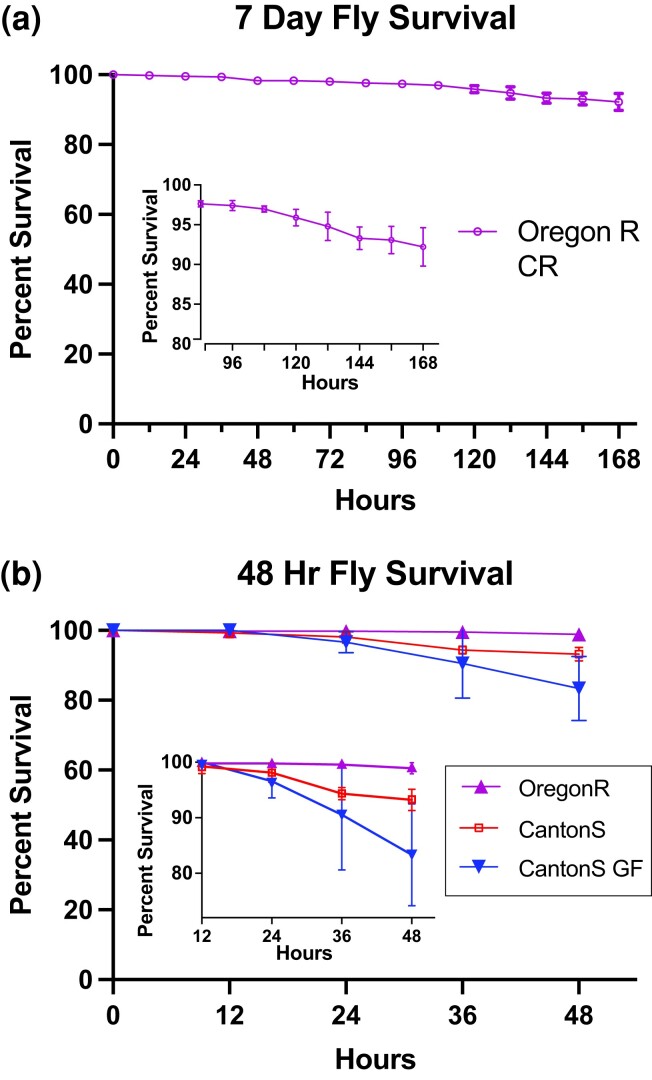
Fly survival in the WAFFL. a) 7 d time course of fly survival in WAFFL with liquid 10:5 food using conventionally reared OregonR flies. Inset depicts enlarged area. N=311. b) 48 h time course of fly survival in WAFFL with liquid 10:5 food using Conventionally reared OregonR (N=456), Conventionally reared CantonS (N=264), and germ-free CantonS (N=264). Inset depicts enlarged area to better visualize differential data sets.

Fly survival in the WAFFL is critical to any assay and indicates the level of stress on the flies. To optimize fly survival in the WAFFL, we iteratively improved conditions using survival assays. Initially, female survival at 24 h was 86.5%, N=288, SEM=2.2. Male survival was 87.5%, N=288, SEM=2.96. At 48 h, survival in the WAFFL had decreased to 57.8% and 46.1% for females and males, respectively. In an attempt to improve survival, we changed the food at 24 h, resulting in a slight increase in total fly survival at 48 h to 63.2% female and 50.6% male (Fig. 2b). However, with the transfers, many flies died from getting squished. We developed the transfer adapters to alleviate this problem (Fig. 1d). Flies also still appeared to die due to dehydration despite the food transfers. We developed high humidity conditions (80–90% RH) by enclosing the WAFFL in a large plastic box with a saturated salt solution at the bottom. After these improvements, survival in the WAFFL was equivalent to survival in deep 96-well plates with solid agar food (Supplementary Fig. S3). Similar average survival at 24 h was observed in both sexes (85–87.5% survival, N=288, SEM=5.82−12.8).

Of the flies that died, a common cause of death was dehydration due to the capillaries at the bottom of WAFFL well coming into contact with an air bubble. We took greater care to prevent air bubbles in the food and visually ensured that each well got an adequate food supply. We also wrapped the base of the WAFFL with Parafilm to decrease desiccation of the wells at the edge of the plate. Through many iterations of conditions, we achieved long-term survival at greater than 90% for 1 week (N=311, Fig. 2a). We additionally assayed fly survival in the WAFFL at 48 h for different fly genotypes. OregonR flies had the best survival, followed by CantonS flies and last CantonS flies raised germ-free (Fig. 2b), indicating slight differences in the WAFFL may be due to fly robustness, with germ-free flies being the least robust. Flies in 96-well plates on solid food had similar survival. Overall, we find that the WAFFL is effective for delivering food to many flies for at least a week.

### Fly feeding rate in the WAFFL varies by food type

**Fig. 3.**
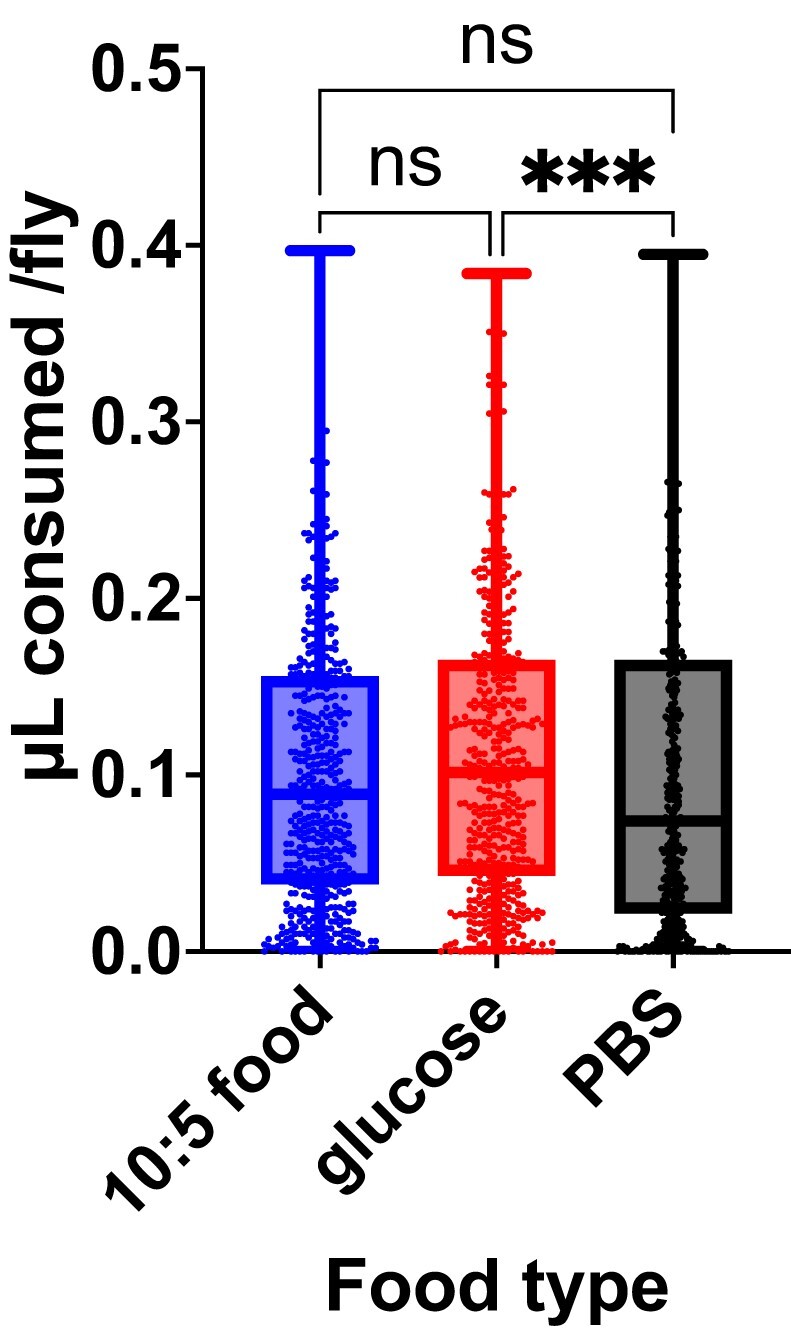
Fly feeding rate in the WAFFL by food type. A modified EX-Q assay using liquid food in the WAFFL was used to determine fly feeding behavior, comparing 10:5 food (N=538), 10% glucose (N=506), or 1× PBS (N=502). Mann–Whitney test (P=0.013) with pairwise Wilcoxon rank sum between each pair. Dunn’s correction for α. ***P < 0.001.

Dose is a carefully controlled parameter in drug screens. To measure the dose of food that flies consumed in the WAFFL, we adapted the Con-Ex (Shell *et al.* 2018) and EX-Q (Wu *et al.* 2020) approach, where consumption is measured based on excretion of a dye in the food, to the WAFFL format (see “Materials and methods”). Flies were first deprived of food for 4 h where they were provided water. Individual flies were then fed on food containing blue dye for 2 h followed by 4–6 h on food without dye to allow the blue dye to pass through the digestive tract and collect it for quantification. By resuspending the frass in 1× PBS and comparing the absorbance at 630 nm to a standard curve, we were able to quantify the amount of food ingested per fly. On 10:5 food, CantonS flies consumed 0.10 μL of food per fly in 2 h, or 0.05 μL per hour (N=538 flies, SEM=0.004, Fig. 3, which is similar to published measurements (Ja *et al.* 2007; Deshpande *et al.* 2014). To test whether diet affected food consumption in these conditions, we repeated the assay with 10% glucose and 1× PBS. On 10% glucose, flies consumed 0.11 μL per fly in 2 h (N=506 flies, SEM=0.004, Fig. 3). On 1× PBS, flies consumed 0.099 μL per fly in 2 h (N=502, SEM=0.004, Fig. 3), indicating small effects of diet on food consumption at short time scales after food deprivation. These results indicate flies can be dosed in the WAFFL and the amount consumed per fly can be quantified, which is important for drug studies.

### Video monitoring

There are many fly behavior monitoring systems (Simon and Dickinson 2010; Kohlhoff *et al.* 2011; Gilestro 2012), however, these are not designed to perform an assay with 96 flies and simultaneously record high resolution video, while also being easily portable and compatible with other 96-well equipment. Therefore, we developed a new video system for the WAFFL.

**Fig. 4.**
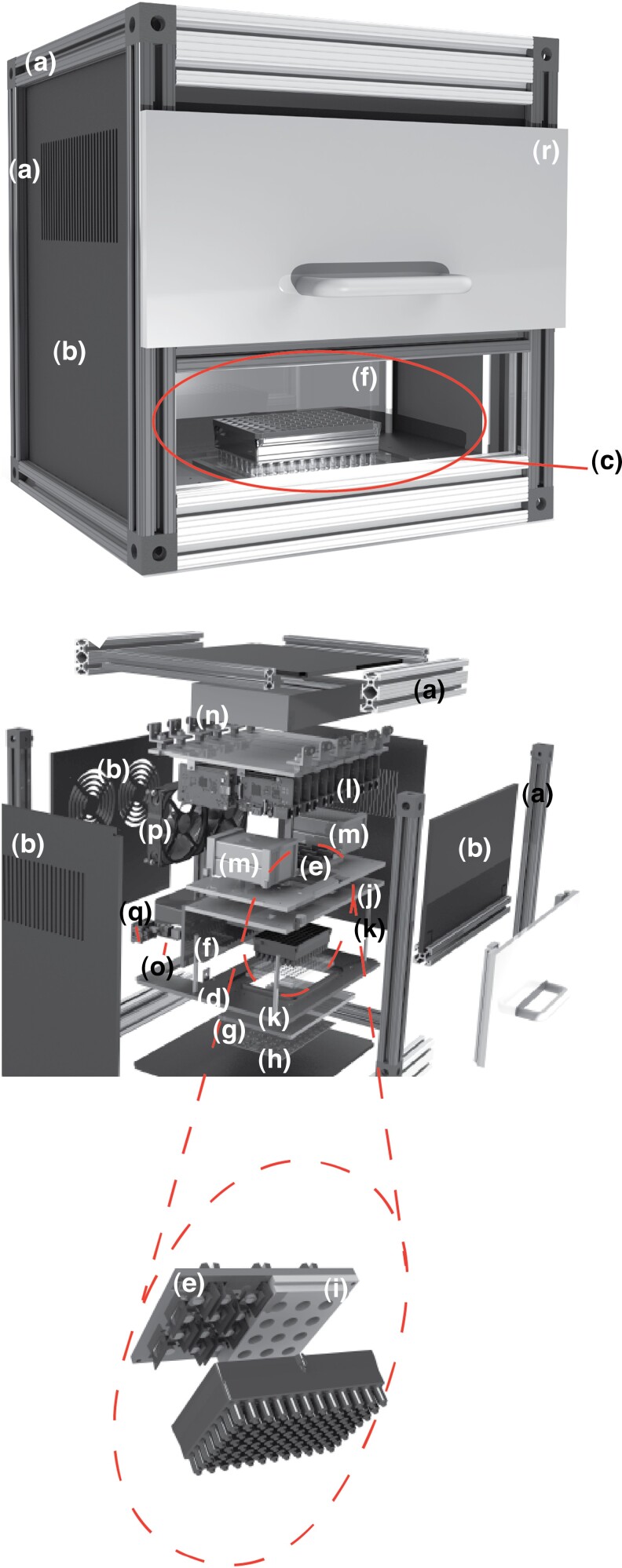
MUFFIN video recording system. Exploded diagram of the MUFFIN. a) Slotted aluminum frame, b) acrylic covers, c) monitoring chamber, d) acrylic base plate, e) camera sensors and lenses, f) acrylic wall, g) mounting cutout with NIR light (700 m) diffuser, h) printed circuit board, i) 3D printed camera board mount, j) acrylic support, k) support stand-offs, l) 24 RPI modules, m) USB power ports for RPIs, n) 24-port Ethernet switch and a 5-port Ethernet switch, o) output cable, p) fans, q) power entry module, and r) sliding lift door.

The MUFFIN was designed to capture high resolution (720p) and frame rate (30 fps, frames per second) video for each chamber in the WAFFL to track fly position and enable detection of certain behaviors of interest (Fig. 4, Supplementary Fig. S4). Aside from the image resolution requirement, practical constraints for the MUFFIN design were mainly compactness and cost-effectiveness. The MUFFIN employs 24 RPI cameras fitted with standard lenses, each of which was focused on a grid of 4 wells (Supplementary Fig. S4). This number of cameras allows capture of the field of view with sufficient resolution in a small working distance, allowing for a compact system.

To avoid compromising the results of MUFFIN experiments with confounding factors introduced by the system components (such as heat and noise), and to protect the electronics from the corrosive volatile hydrocarbons that flies produce, the MUFFIN was built with a recording chamber physically isolated from the rest of the system’s electronics and components. The recording chamber was also separated from the external environment with a pull-down door (Fig. 4f). The recording arena has a keyed plate to hold the WAFFL directly underneath the system cameras, such that each camera’s field of view was fixed and consistent between recording sessions (Supplementary Fig. S4). Each of the 24 RPI’s streamed video to a local computer via a local gigabit network. We saved video on a computer and watched the progress of the live recording by streaming. The MUFFIN GUI for using the software of the MUFFIN is shown in Supplementary Fig. S4.

For experiments in the MUFFIN, we placed a thin clear acrylic plate cover on the WAFFL to prevent flies from escaping without obstructing the cameras; view (Fig. 1, Supplementary Fig. S1). We tested several clear acrylic covers of different thicknesses varying from 1.59 to 6.35 mm, to choose the one resulting in the highest image quality. Thicker acrylic pieces resulted in darker images, and thinner acrylic resulted in distortion due to flexing. We chose the 3.18 mm thickness piece for further experiments.

We placed the MUFFIN inside the same incubator as the WAFFL experiments to make the conditions consistent. In our current work, the conditions were an average temperature of 25°C and average humidity of 75%. The temperature inside the MUFFIN was up to 27°C due to heat generated by electric parts of the device. We chose a high humidity to avoid evaporation of the liquid media due to its low volume of 16 μL. Having a higher humidity also prevented condensation of the liquid (probably from evaporating food) on the acrylic cover, which obscured filming.

The chosen MUFFIN cameras were versatile in recording resolution and acquisition rate. We have developed a first generation set of custom algorithms that detect fly silhouettes in the image and identify the centroid of the silhouette of the fly. The silhouette can be used to compute a measure of the frequency of interactions with food, while the centroid is used to compute a measure of the locomotive activity of the fly in terms of distance traveled in the X–Y axis, although the software does not provide estimates for Z-axis position.

### Fly feeding time course

**Fig. 5.**
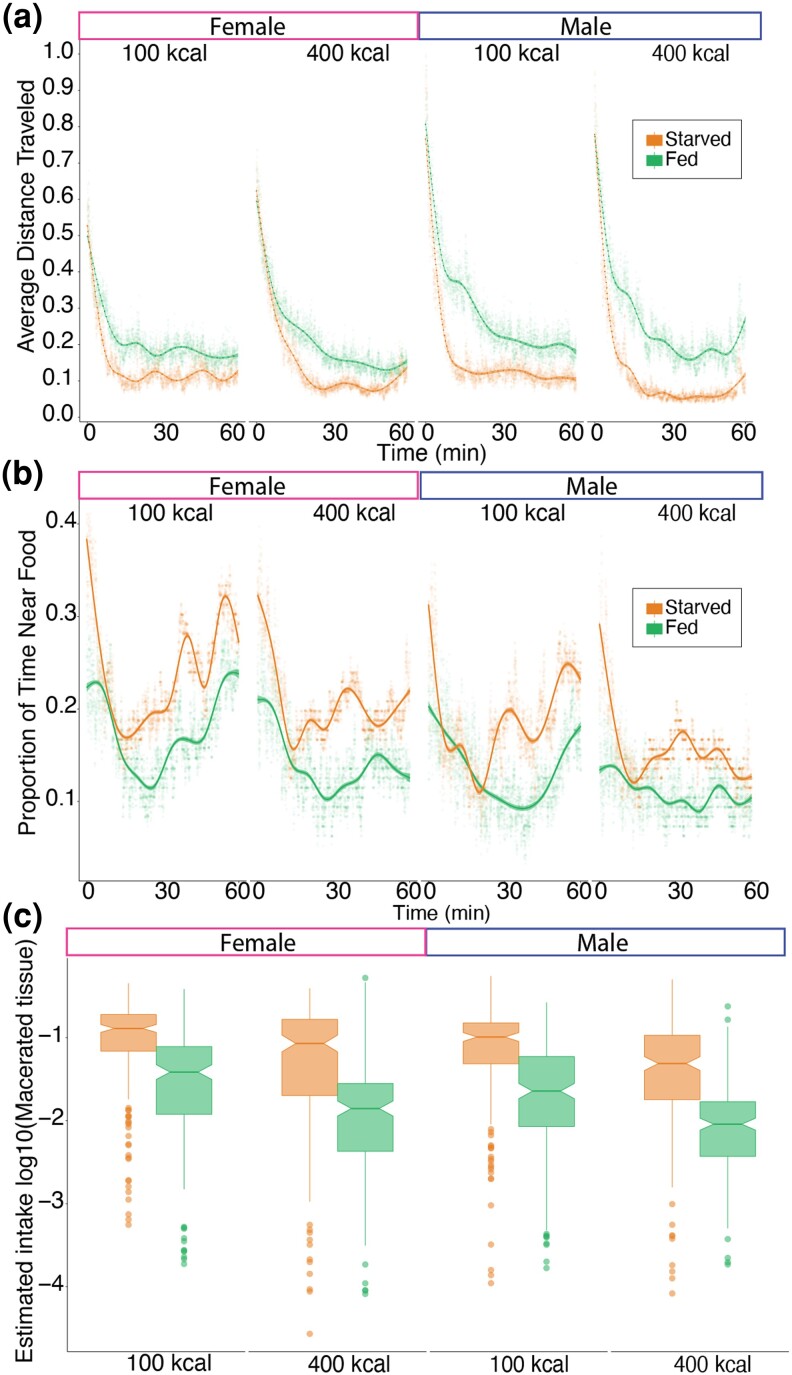
Fly feeding behavior using the MUFFIN. Plotted smoothed data collected with the MUFFIN, compared females to males, fed flies versus starved, and flies provided a high- versus low-caloric content diet a) Average distance travel within 1 h of recording. Each point along the smoothed trend line is a fly averaged across 1 s. Because z-movements are not tracked, the distance is given in relative units normalized to the maximum distance observed in a time step. b) Proportion of time near the food, defined as the proportion of frames for every 2 min of video in which the fly was in the food region circle (as defined in “Materials and methods”). Each point along the smoothed trend line is a fly averaged across 1 s. c) Box plots showing the measurement of relative food content per fly after a 1 h feeding with CDF with sulforhodamine B dye using the WAFFL (as defined in “Materials and methods”).

To correlate the EX-Q feeding data with behaviors of the flies, we video recorded flies feeding in the MUFFIN system. We made 3 measurement types: distance traveled, time spent at feeding ports, and food within the gastrointestinal tract. We evaluated 3 experimental variables: pre-fed versus pre-starved, high (400 kCal) versus low (100 kCal) calorie food offered, and sex. As described in the methods, both sexes were included in each plate, and the pre-fed or pre-starved treatments were randomized in the plates to control for position artifacts. Flies were exposed to these pretreatments for 6 h. In parallel, 96 flies per plate were exposed to the same caloric diet while recording in the MUFFIN. Experiments were replicated 4 times for each caloric diet; a total of 768 flies were recorded during 24 h for these experiments (∼83 million frames). We focus on the first hour of recording since in our preliminary experiments we observed that 95% of pre-starved flies fed within the first hour (Fig. 5).

Overall, pre-fed flies on average traveled more distance than pre-starved flies (p<0.001, Fig. 5a, Supplementary Fig. S5). We observed that pre-starved flies that were fed with a calorically dense 400 kcal/L diet on average traveled less than flies fed 100 kcal/L food (p<0.005, Fig. 5a, Supplementary Fig. S6). Thus, even in the first hour of feeding, a higher calorie meal results in lower average distance traveled. Previous studies have shown that larger, denser meals cause lower activity in flies (Murphy *et al.* 2016). Pre-fed flies traveled similar average distances within the first hour of recording, regardless of the caloric content of the food (Fig. 5b, Supplementary Fig. S6b). Comparing males to females, we found no difference in average distance traveled between sexes at 1 h, but fed males traveled more than fed females in the first 20 min (Fig. 5b, Supplementary Fig. S7a). These results demonstrate that we can measure locomotion behavior changes due to pre-treatments, diet caloric content, and sex.

To determine how much time flies spent at the feeding ports, we used a circular area of interest at that location and measured time within that area as described in the Materials and methods section. Part of the reduced motility of fed pre-starved flies was due to the fact that the pre-starved flies spent significantly more time on average at the food than the pre-fed flies (p<0.05, Fig. 5b, Supplementary Fig. S5b). Additionally, the flies consuming the higher caloric diet spent less time on average at the food in later time points, suggesting that they may have been satiated (p<0.005, Fig. 5b, Supplementary Fig. S6b).

Some of our results suggested that there may be differences in the bolus size of a fly meal in our experiments. For example, pre-fed flies did not show significant differences in travel average distance due to the caloric content of the food, while pre-starved flies did (Fig. 5a, Supplementary Fig. S5a). To see if we could measure the food content within a fly, we measured food consumption by the flies as assayed with a fluorescent dye, sulforhodamine B. After a 1 h of feeding we crushed the flies, to assay the relative fluorescence on a plate reader (Fig. 5c). We showed earlier (Fig. 5b) that pre-starved flies spend more time at the feeding port area, and the food content of pre-starved flies was greater than pre-fed flies ($p<10^{-10}$), suggesting that they spent that additional time actively feeding. We also observed that the flies fed on lower calorie food had more food in their bodies than those that consumed high calorie food, suggesting that flies may compensate for lower food quality by eating more. We did not observe a major difference in food consumption between the sexes (Fig. 5c, Supplementary Fig. S7).

This experiment shows the potential of using the WAFFL and MUFFIN to study different factors in a large number of individual flies, which could translate to the identification of phenotypes in response to a drug treatment or genetic alteration. Using the MUFFIN, we were also able to infer that the food consumption we measured by EX-Q primarily occurred in the first 15 min after food exposure. These tools will allow the field to address a broad range of biological questions in *Drosophila* that were previously inaccessible due to the lack of high-throughput tools to easily manipulate and treat individual flies.

## Discussion

We have provided a detailed description of the WAFFL and its complementary component, the MUFFIN. In conjunction, these devices provide a comprehensive system that makes plausible the performance of high-throughput pharmacological screens that include the monitoring of adult flies (Fig. 4).

**Fig. 6.**
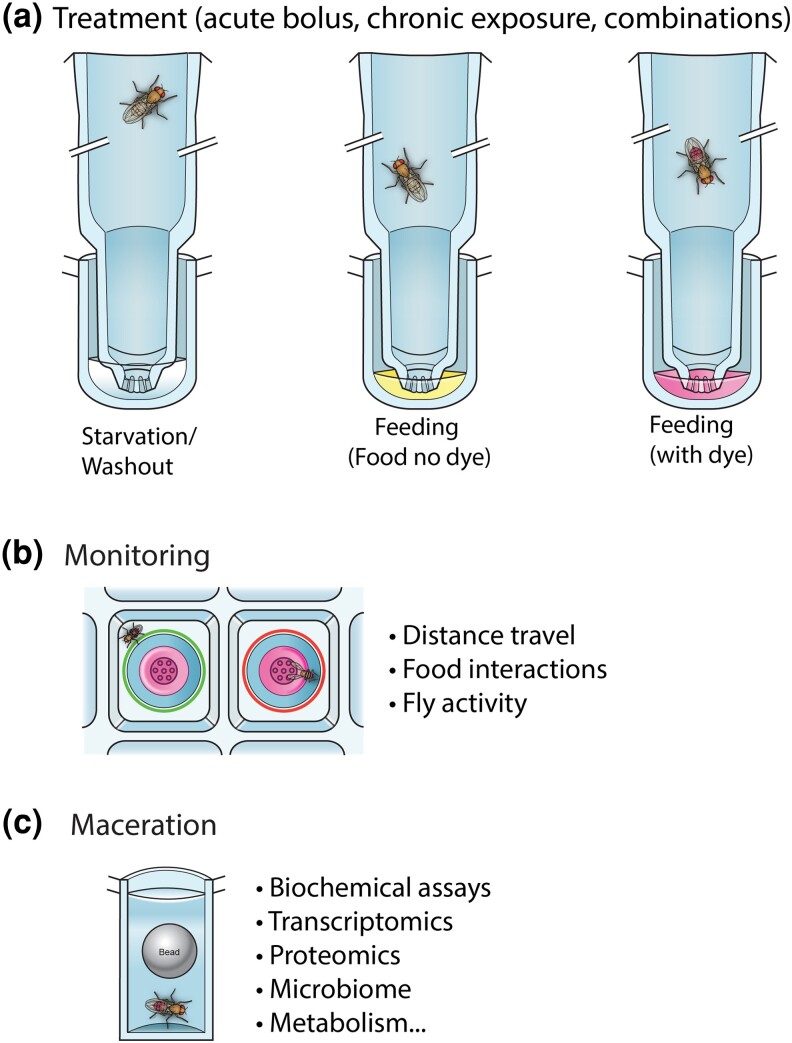
WAFFL overview. a) Flies are treated individually in wells with small amounts of food. b) Flies are monitored while feeding. c) Flies can be macerated for downstream analysis.

The WAFFL system allows performance of a series of assays with the same set of flies, including food consumption, excretion, and fly physiological assays on fly extract (Fig. 6a). Furthermore, individual fly behavior including activity and food interactions can be monitored by the MUFFIN, allowing a broad range of single fly phenotypes to be correlated (Fig. 6b). Having the flexibility to change the food within seconds allows studies aimed at understanding the effects of acute exposure versus chronic exposures as well as determining the effects of the order and timing of delivery of different diets and drugs to the flies.

Treated flies can be easily collected after treatment and evaluated individually by microscopy or tissue homogenization for assays such as transcriptomics, proteomics, and metabolite measurements (Fig. 6c). The combination of assays increases the number of measurements per fly, allowing the discrimination of correlated sources of variation. Using manual and semi-automated protocols, we have performed up to 20,000 fly experiments in a week. The only serious impediment to screening we observed was evaporation of liquid media when performing long assays (>12 h) or when heat shock or elevated temperature is part of the experimental design. Evaporation is a common problem in essentially all high-throughput assays on cell-based screens (Maddox *et al.* 2008; Turner *et al.* 2008; Das *et al.* 2016; Knight *et al.* 2018). However, a major advantage of the WAFFL is that one can simply replace the media by transferring the WAFFL to a fresh feeding plate in longer assays.

While the one-fly, one-treatment-per-well design is a strength in many settings, there may be situations in which a researcher wants to co-house flies or offer flies a choice of media. The WAFFL allows up to 3 flies per well, the design could be modified to increase well size or, e.g. merging 2 or 4 top square sections of the well would permit housing more flies per well and providing a choice of foods, which could be tracked using different dyes. One might also want to reduce the z-dimension to create an arena rather than a deep well. This could be especially useful for complex behavior assays.

In summary, we developed a comprehensive system that can support both parallel and sequential assays in adult flies using the same platform. We envision that the WAFFL system has the potential to revolutionize the use of phenotypic screening in *Drosophila* and inspire the development of related systems for other small model organisms.

## Supplementary Material

jkad012_Supplementary_Data

## Data Availability

Data are deposited at Dryad. https://doi.org/10.5061/dryad.q83bk3jmt Supplementary material are available at G3 online.
