# Supplementary material for: Whole Animal Feeding FLat (WAFFL): a complete and comprehensive validation of a novel, high-throughput fly experimentation system
Source: G3 (Bethesda). 2023 Jan 18;13(3):jkad012. doi: 10.1093/g3journal/jkad012 (PMC9997563; doi:10.1093/g3journal/jkad012)
Supplement: jkad012_Supplementary_Data [file jkad012_supplementary_data.pdf]

OXFORD
G3 Genes | Genomes | Genetics

Supplemental Material for: Whole Animal Feeding FLat (WAFFL): a complete and comprehensive validation of a novel, high-throughput fly experimentation system

Maria D.L.A. Jaime[1,2], Ghadi H. Salem[3], Daniel J. Martinez[1], Sean Karott[2], Alejandra Flores[2,4], Cameron D. Palmer[2], Sharvani Mahadevaraju[2,5], Jonathan Krynitsky[3], Marcial Garmendia-Cedillos[3], Sarah Anderson[3], Susan Harbison[6], Thomas J. Pohida[3], William B. Ludington[*,1,7] and Brian Oliver[*,2,5]

[1]Department of Embryology, Carnegie Institution of Washington, Baltimore, MD 21218 USA
[2]Laboratory of Cellular and Developmental Biology, National Institute of Diabetes and Digestive and Kidney Diseases, National Institutes of Health, 50 South Drive, Bethesda MD 20814 USA
[3]Instrument Development and Engineering Application Solutions, National Institute of Biomedical Imaging and Bioengineering, National Institutes of Health, 12 South Dr, Bethesda, MD 20892
[4]Department of Physiology and Biophysics, Case Western Reserve University, 10900 Euclid Ave, Cleveland OH 44106
[5]Laboratory of Biochemistry and Genetics, National Institute of Diabetes and Digestive and Kidney Diseases, National Institutes of Health, 50 South Drive, Bethesda MD 20814 USA
[6]Laboratory of Systems Genetics, National Heart Lung and Blood Institute, National Institutes of Health, 10 Center Drive, Bethesda MD 20814 USA
[7]Department of Biology, Johns Hopkins University, Baltimore, MD 21218 USA.

[*,1,2,8]Corresponding authors: Department of Embryology, Carnegie Institution of Washington, Baltimore, MD 21218 USA. ludington@carnegiescience.edu
Laboratory of Cellular and Developmental Biology, National Institute of Diabetes and Digestive and Kidney Diseases, National Institutes of Health, 50 South Drive, Bethesda MD 20814 USA. briano@nih.gov

Abstract

Non-mammalian model organisms have been essential for our understanding of the mechanisms that control development, disease, and physiology, but they are underutilized in pharmacological and toxicological phenotypic screening assays due to their low throughput in comparison with cell-based screens. To increase the utility of using *Drosophila melanogaster* in screening, we designed the Whole Animal Feeding FLat (WAFFL), a novel, flexible, and complete system for feeding, monitoring, and assaying flies in a high-throughput format. Our 3-D printed system is compatible with inexpensive and readily available, commercial 96-well plate consumables and equipment. Experimenters can change the diet at will during the experiment and video record for behavior analysis, enabling precise dosing, measurement of feeding, and analysis of behavior in 96-well plate format.

Keywords: Drosophila; High-throughput; Feeding Behavior; Screening

© The Author(s) 2023.

Supplemental figures and tables

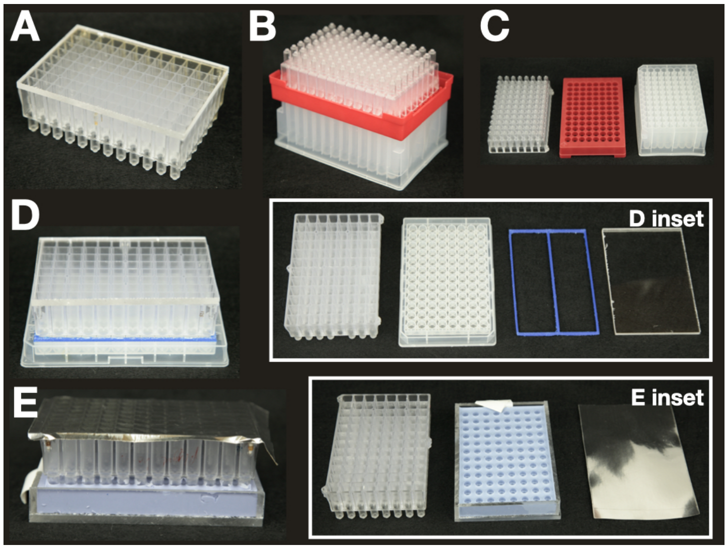

Figure 1 Supplemental images of WAFFL parts. (A) WAFFL. (B) WAFFL transfer assembly. (C) WAFFL transfer assembly parts. (D) WAFFL feeding assembly with clear acrylic top. (D inset) Parts for WAFFL feeding assembly. (E) Frass recovery setup for EX-Q feeding. (E inset) parts for EX-Q frass recovery.

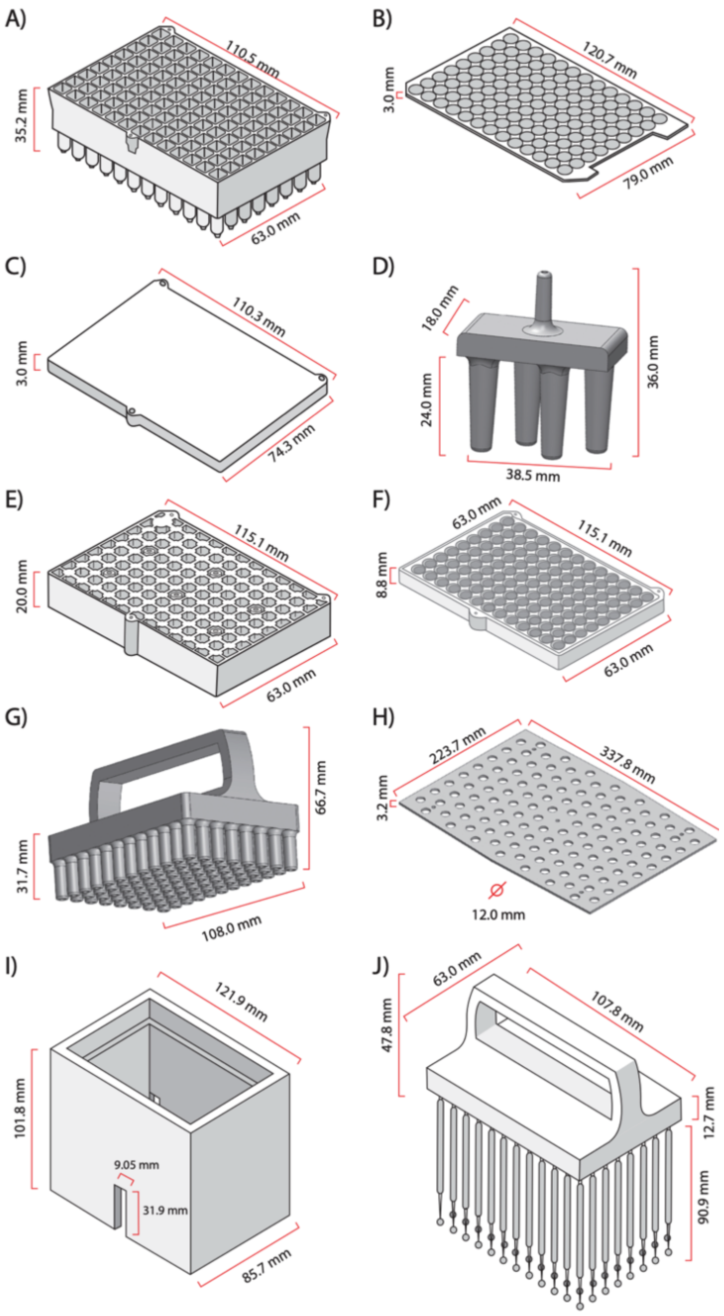

Figure 2 Supplemental additional WAFFL tools. (A) WAFFL. (B) Silicone mat. (C) Clear acrylic top. (D) Four fly loading vacuum device. (E) Transfer temporary collection plate. (F) Transfer adapter. (G) 96 well remover of stainless steel maceration beads. (H) Rectangular base plate for stabilizing the WAFFL in the incubation chambers. (I) IERN box. (J) WAFFL Insect Excrement Removal Nano-brushes (WAFFL-IERN).

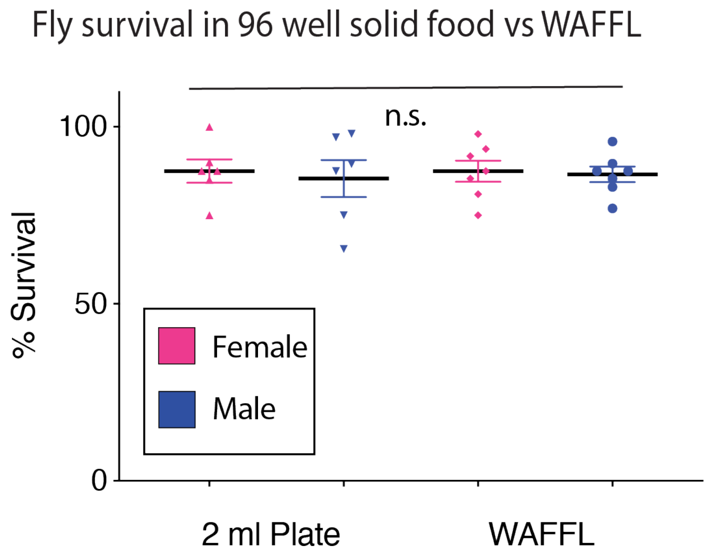

Figure 3 Supplemental fly survival in the WAFFL. 24 h time course of fly survival in WAFFL with liquid CDM food versus in a 96 well plate with solid agar Bloomington diet. Males and females were assayed. Results from 6 independent WAFFLs, with 48 male and female flies per treatment. Mean and SEM of the percentage survival for each plate.

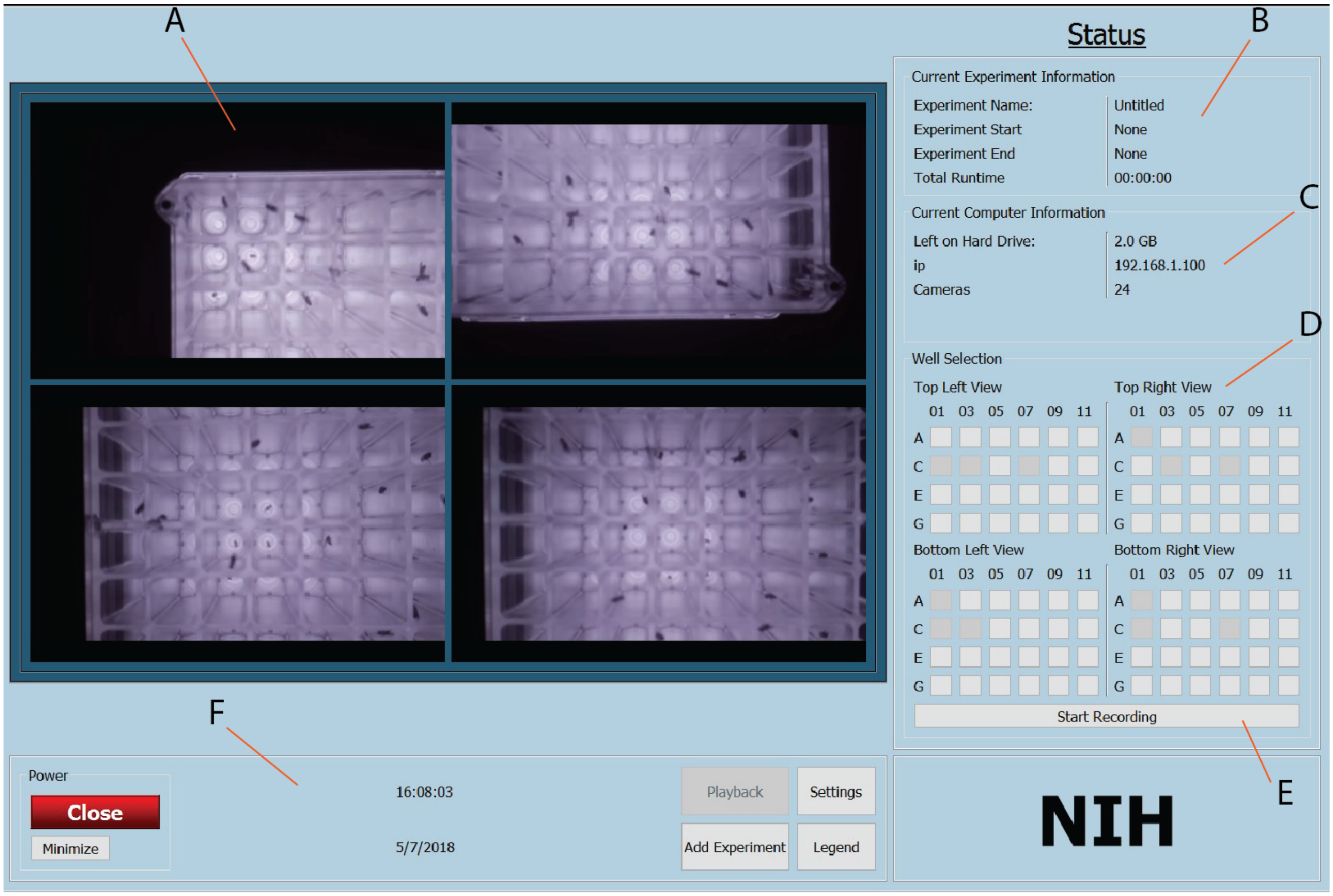

Figure 4 Supplemental MUFFIN GUI software interface. The graphical user interface (GUI) of the MUFFIN system displays all the cameras being used to record the flies. It is composed of the (A) Video display window, (B) Experiment status indicators, (C) Computer information section, (D) Well selection area, (E) Manual stop and start recording button, and (F) the program settings control panel with date and time.

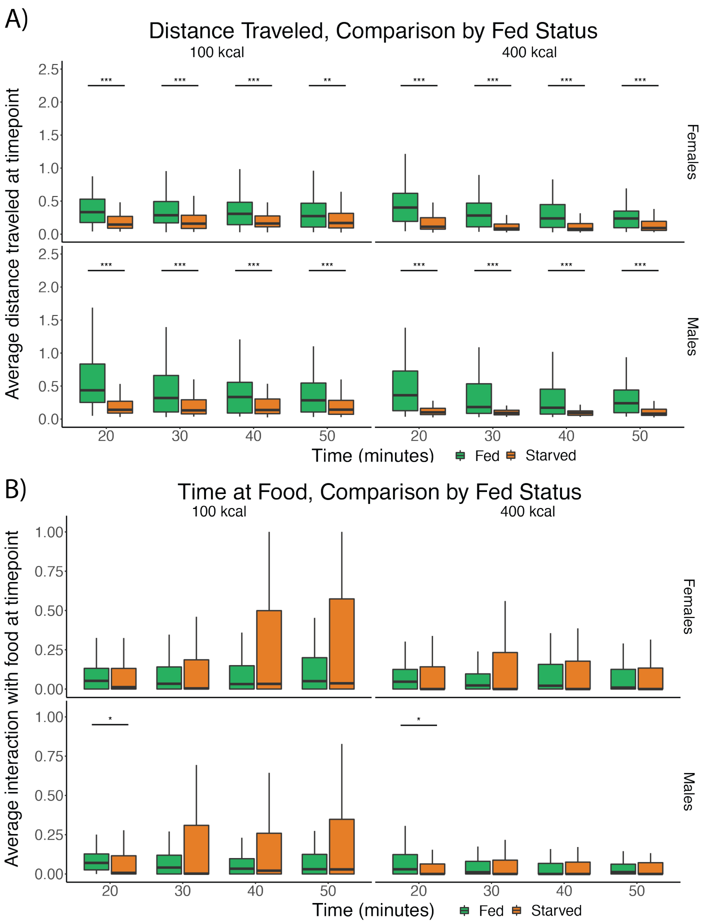

Figure 5 Supplemental MUFFIN data analyzed by fed vs starved status. Muffin recorded raw data divided in 10 min windows (15- 55 min). (A) Box plots showing the distance travel between fed and starved flies. (B) Box plots showing the time fed and starved flies interact with the food. A two tailed Wilcoxon signed rank (Mann Whitney U) tests on pairs of data distributions and Benjamini & Hochberg false discovery rate correction was applied in 10 min windows for distance traveled and food interactions. *P < 0.05, **P < 0.005, ***P < 0.001

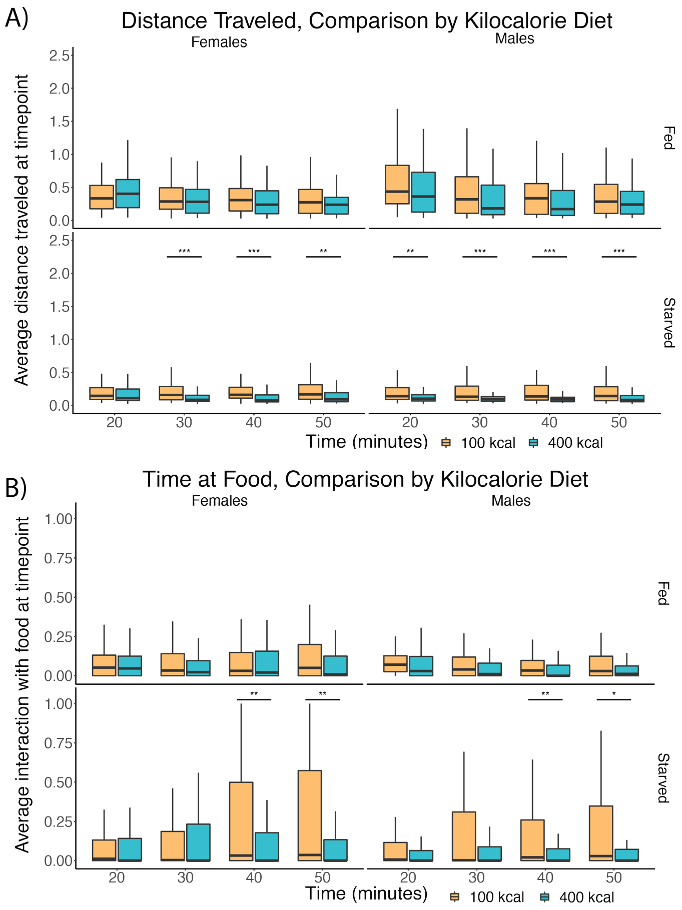

Figure 6 Supplemental MUFFIN data analyzed by fed vs starved status. Muffin recorded raw data divided in 10 min windows (15 – 55 min). A) Box plots showing the distance travel by flies fed with 100 kCal or 400 kCal diet. B) Box plots showing the time flies expend at the 100 kCal or 400 kCal food. A two tailed Wilcoxon signed rank (Mann Whitney U) tests on pairs of data distributions and Benjamini & Hochberg false discovery rate correction was applied in 10 min windows for distance traveled and food interactions. *P < 0.05, **P < 0.005, ***P < 0.001

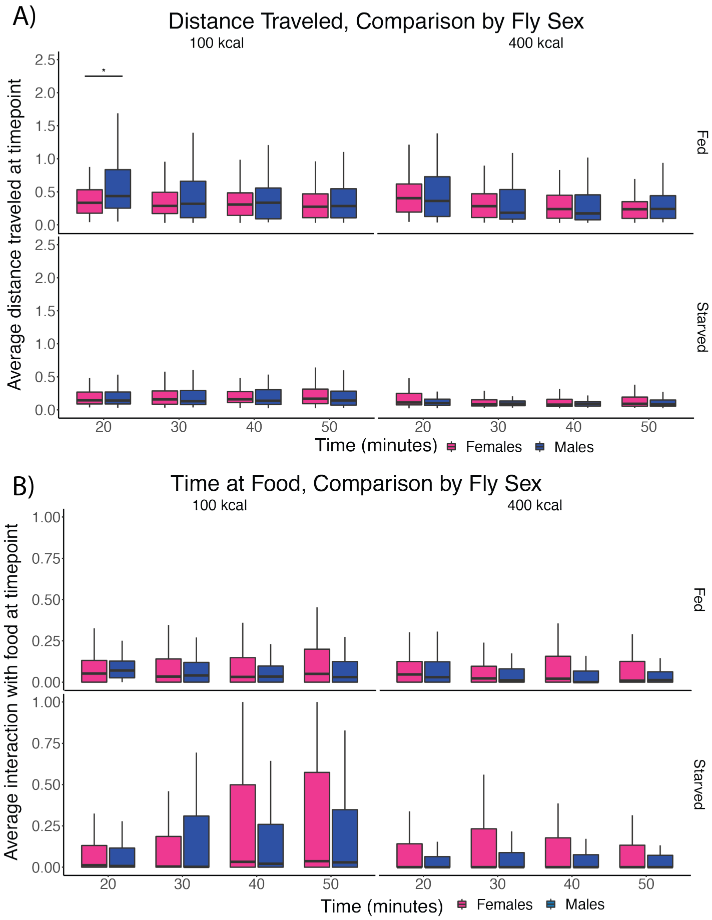

Figure 7 Supplemental MUFFIN data analyzed by fed vs starved status. Muffin recorded raw data divided in 10 min windows (15 – 55 min). (A) Box plots showing the distance travel by female and male flies. (B) Box plots showing the time female and male flies expend at food. A two tailed Wilcoxon signed rank (Mann Whitney U) tests on pairs of data distributions and Benjamini & Hochberg false discovery rate correction was applied in 10 min windows for distance traveled and food interactions. *P < 0.05, **P < 0.005, ***P < 0.001
